# Cervical Paraganglioma Mimicking Thyroid Nodule: A Rare Clinical Case

**DOI:** 10.1155/2016/8527279

**Published:** 2016-03-15

**Authors:** Berna İmge Aydoğan, Serpil Dizbay Sak, Sevim Güllü

**Affiliations:** ^1^Faculty of Medicine, Department of Endocrinology and Metabolic Diseases, Ankara University, 06410 Ankara, Turkey; ^2^Faculty of Medicine, Department of Pathology, Ankara University, 06410 Ankara, Turkey

## Abstract

*Objective*. Paraganglioma is a rare neuroendocrine tumor. When it is located in the neck, it is commonly misdiagnosed as other thyroid neoplasms.* Case Report*. We report a case of cervical paraganglioma in a 55-year-old female. Patient was admitted to our clinic with goiter and neck pain. Thyroid ultrasonography revealed a 20 mm solitary, heterogeneous nodule located in the upper pole of left thyroid lobe. Fine needle aspiration cytology was nondiagnostic. She underwent left lobectomy and histopathology showed paraganglioma.* Discussion*. Cervical paragangliomas should be considered in the differential diagnosis of thyroid nodules.

## 1. Introduction

Paraganglioma (PGL) is an uncommon neuroendocrine tumor which originates from primitive neural crest of autonomic nervous system. Head and neck PGLs account for 3% of all PGLs [1]. Carotid body tumors are the most common PGLs (60%) of the skull base and neck [2]. Vagal, jugulotympanic, and laryngeal paraganglia are the exceptional localization. Thyroid gland and adjacent tissue are rare localization of PGLs, as paraganglia are not present in the thyroid tissue. They seem to originate from inferior laryngeal paraganglia which may rarely be located within the thyroid capsule [3].

Both primary thyroid PGLs and cervical PGLs mimicking thyroid nodules have been reported in the literature. These tumors cause diagnostic challenge. Neck PGLs are usually nonfunctional and if located within or near to the thyroid gland, they usually present as a slightly enlarging, solid, hypervascular nodule of the thyroid gland [3].

Fine needle aspiration biopsy is commonly nondiagnostic in these tumors [4]. PGLs can be misinterpreted as medullary thyroid cancer (MTC) because of their middle-upper pole localization within the lobes of the gland and microscopic features on cytology.

## 2. Case

**Figure 1:**
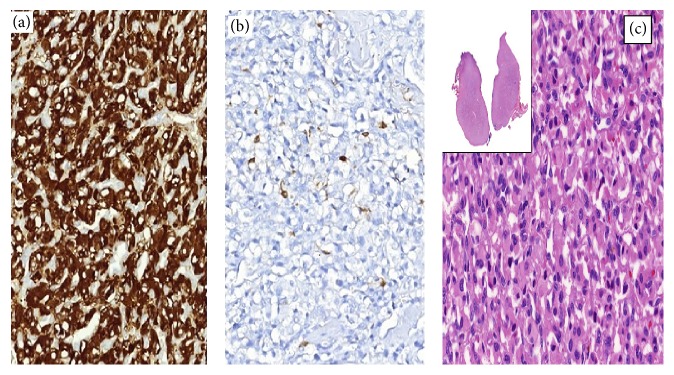
Tumor cells staining with synaptophysin (a), sustentacular cells staining for S100 (b), and nodule consisting of nests of eosinophilic polygonal cells (hematoxylin and eosin) (c).

Our patient was a 54-year-old female who presented with neck swelling. She had a multinodular goiter and was admitted to our outpatient clinic for thyroid fine needle aspiration biopsy. Ultrasonography revealed four nodules located in the left lobe of thyroid gland; three of them were smaller than 5 mm and one was 20 mm in diameter. The greater nodule was heterogeneous and hypervascular and located in the upper pole of left thyroid lobe. Patient was asymptomatic except that she described tenderness during the palpation of the nodule. Thyroid function tests were within the normal ranges. Antithyroglobulin and antithyroid peroxidase antibodies were undetectable. Because of the upper pole localization and heterogeneous morphology of the nodule, ultrasonographic appearance resembled medullary thyroid carcinoma but calcitonin (5.7, normal range; 0–10 pg/mL) and CEA (1.02, N; 0–3 ng/mL) measurements were found normal. Fine needle aspiration biopsy was performed with 25-gauge needle and cytology was reported to be nondiagnostic. Because of the suspicious ultrasonographic features of the nodule, she underwent left lobectomy. Gross examination showed a well circumscribed solid nodule of 20 mm, attached to the upper pole of left lobe with a fibrous band. On histopathological examination, a vascular tumor with trabecular/insular organization, consisting of polygonal cells with abundant granular eosinophilic cytoplasm, was observed (Figure 1). Immunohistochemistry showed strong expression of CD56, chromogranin A, and synaptophysin. Sustentacular cells were highlighted by S100 protein (Figure 1). Tumor was completely negative for thyroglobulin, calcitonin, TTF 1, HBME1, CK19, Galectin-3, parathormone, and desmin.

Postoperative Ga68-DOTATE scan showed no pathological uptake. Urinary catecholamine levels were normal. No recurrence has been observed in 30 months of follow-up.

## 3. Discussion

Head and neck region is a common location for extra adrenal PGLs. These tumors present as a slowly growing mass of neck and may also be located in thyroid capsule. Both primary thyroid PGLs located in thyroid capsule and cervical PGLs mimicking thyroid nodules have been reported in the literature [5–8]. High proportions of these tumors were reported in middle aged females just as our case [8]. Normal thyroid gland does not involve paraganglia but inferior laryngeal paraganglia are thought to be the origin of thyroid PGLs.

Preoperative diagnosis of both thyroid PGLs and PGLs mimicking thyroid nodules is always challenging. Ultrasonographic features are not specific, as hypervascular nodules of thyroid are not rare and localization of tumor does not provide favorable information for differential diagnosis.

Fine needle aspiration biopsies of these tumors are usually nondiagnostic. PGLs of thyroid gland are frequently confused with MTC because of the similarity of microscopic and some immunohistochemical features [9]. However, cytokeratin, calcitonin, CEA, and TTF1 positivities, which are frequently observed in MTC but not in PGLs, help to distinguish these two different tumor types. Additionally, metastatic carcinoid tumors, Hurthle cell neoplasms, and hyalinizing trabecular tumors of the thyroid may be indistinguishable from PGL, on hematoxylin and eosin stained sections [4]. Immunohistochemical examination is usually required for the diagnosis of PGL, if it is in an atypical localization especially. PGL is typically positive for chromogranin A, synaptophysin, tyrosine hydroxylase, and CD56 and negative for CEA, calcitonin, TTF1, and cytokeratin [10]. Sustentacular cells are decorated with S100 and GFAP. On the other side immunohistochemistry is unable to identify the origin of head-neck PGLs and observation of surgeon is important at this point. In our case, no relation of tumor with vascular structures or laryngeal nerve was observed at surgery. Tumor, which was attached to upper pole of the thyroid, was dissected with left lobe and isthmus. No invasion to the adjacent tissues was observed.

Histopathological features are not efficient for identifying tumor aggressiveness, so only presence of metastases and/or local recurrence confirms a malignant PGL. Thyroid PGLs are not aggressive tumors and for benign neck PGLs 5-year survival is 95% [11]. Total thyroidectomy or lobectomy is the choice of treatment. Previously, Calò et al. demonstrated that the entire reported thyroid PGLs had benign course with no recurrence/metastases [12].

Immunostaining or genetic analysis for succinate dehydrogenases (SDHB, SDHC, and SDHD) is recommended for differential diagnosis of familial PGL syndromes [5, 13]. Unfortunately we could not perform an immunostaining or genetic testing for germline SDH mutation but personal/family history was not suggestive of hereditary syndromes.

In summary, the preoperative diagnosis of thyroid PGL is difficult and it should be considered in the differential diagnosis of thyroid nodules. Medullary thyroid carcinoma (MTC) is the most frequent tumor confused with PGLs of thyroid.

## References

[1] Sykes J. M., Ossoff R. H. (1986). Paragangliomas of the head and neck. *Otolaryngologic Clinics of North America*.

[2] Pellitteri P. K., Rinaldo A., Myssiorek D. (2004). Paragangliomas of the head and neck. *Oral Oncology*.

[3] Brownlee R. E., Shockley W. W. (1992). Thyroid paraganglioma. *Annals of Otology, Rhinology and Laryngology*.

[4] Vodovnik A. (2002). Fine needle aspiration cytology of primary thyroid paraganglioma. Report of a case with cytologic, histologic and immunohistochemical features and differential diagnostic considerations. *Acta Cytologica*.

[5] Zantour B., Guilhaume B., Tissier F. (2004). A thyroid nodule revealing a paraganglioma in a patient with a new germline mutation in the succinate dehydrogenase B gene. *European Journal of Endocrinology*.

[6] Tiong H. Y., White S. A., Roop L., Furness P. N., Nicholson M. L. (2000). Paraganglioma-an unusual solitary nodule of the thyroid. *European Journal of Surgical Oncology*.

[7] Costinean S., Balatti V., Bottoni A., Old M., Croce C., Wakely P. E. (2012). Primary intrathyroidal paraganglioma: histopathology and novel molecular alterations. *Human Pathology*.

[8] D'Angelo F. A., Antolino L., Magistri P. (2013). Primary thyroid paraganglioma: a rare entity affecting middle-aged women. *The American Surgeon*.

[9] Ferri E., Manconi R., Armato E., Ianniello F. (2009). Primary paraganglioma of thyroid gland: a clinicopathologic and immunohistochemical study with review of the literature. *Acta Otorhinolaryngologica Italica*.

[10] Lee S. M., Policarpio-Nicolas M. L. (2015). Thyroid paraganglioma. *Archives of Pathology & Laboratory Medicine*.

[11] Armstrong M. J., Chiosea S. I., Carty S. E., Hodak S. P., Yip L. (2012). Thyroid paragangliomas are locally aggressive. *Thyroid*.

[12] Calò P. G., Lai M. L., Guaitoli E. (2013). Difficulties in the diagnosis of thyroid paraganglioma: a clinical case. *Clinica Terapeutica*.

[13] Baysal B. E., Willett-Brozick J. E., Lawrence E. C. (2002). Prevalence of SDHB, SDHC, and SDHD germline mutations in clinic patients with head and neck paragangliomas. *Journal of Medical Genetics*.

